# Proteolysis of Rab32 by *Salmonella* GtgE induces an inactive GTPase conformation

**DOI:** 10.1016/j.isci.2020.101940

**Published:** 2020-12-15

**Authors:** Sergey Savitskiy, Rudolf Wachtel, Danial Pourjafar-Dehkordi, Hyun-Seo Kang, Vanessa Trauschke, Don C. Lamb, Michael Sattler, Martin Zacharias, Aymelt Itzen

**Affiliations:** 1 Department of Biochemistry and Signaltransduction, University Medical Centre Hamburg-Eppendorf (UKE), Martinistrasse 52, 20246 Hamburg, Germany; 2 Center for Integrated Protein Science Munich (CIPSM), Department Chemistry, Technical University of Munich, Lichtenbergstrasse 4, 85748 Garching, Germany; 3 Physics Department T38, Technical University of Munich, James-Franck-Strasse 1, 85748 Garching, Germany; 4 Institute of Structural Biology, Helmholtz Zentrum München, 85764 Neuherberg, Germany; 5 Chemistry Department, Biomolecular NMR and Center for Integrated Protein Science Munich, Technical University of Munich, 85748 Garching, Germany; 6 Department of Chemistry, Center for Nanoscience (CeNS), NanoSystems Initiative Munich (NIM) and Center for Integrated Protein Science Munich (CIPSM), Ludwig Maximilians-Universität München, Munich Germany; 7 Centre for Structural Systems Biology (CSSB), University Medical Centre Hamburg-Eppendorf (UKE), Hamburg, Germany

**Keywords:** Molecular Structure, Microbiology

## Abstract

Rab GTPases are central regulators of intracellular vesicular trafficking. They are frequently targeted by bacterial pathogens through post-translational modifications. *Salmonella typhimurium* secretes the cysteine protease GtgE during infection, leading to a regioselective proteolytic cleavage of the regulatory switch I loop in the small GTPases of the Rab32 subfamily. Here, using a combination of biochemical methods, molecular dynamics simulations, NMR spectroscopy, and single-pair Förster resonance energy transfer, we demonstrate that the cleavage of Rab32 causes a local increase of conformational flexibility in both switch regions. Cleaved Rab32 maintains its ability to interact with the GDP dissociation inhibitor (GDI). Interestingly, the Rab32 cleavage enables GDI binding also with an active GTP-bound Rab32 *in vitro*. Furthermore, the Rab32 proteolysis provokes disturbance in the interaction with its downstream effector VARP. Thus, the proteolysis of Rab32 is not a globally degradative mechanism but affects various biochemical and structural properties of the GTPase in a diverse manner.

## Highlights

• Cleavage by GtgE disrupts Rab32 interswitch region and blunts its binding to VARP
• Cleaved Rab32 can bind GDI in both nucleotide states
• Proteolytic PTM of Rab32 results in increased flexibility of the switch regions
• Proteolysis forces Rab32 to its inactive structural state even upon GTP binding

## Graphical abstract

**Figure:**
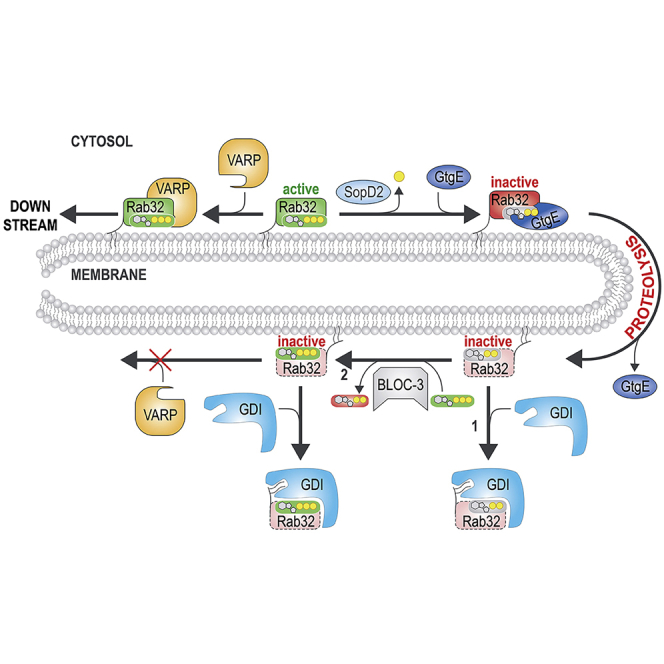

## Introduction

Small GTPases act as important players in intracellular signaling in eukaryotes. The binary switching of these proteins is realized by binding to different guanosine nucleotides: in the guanosine diphosphate (GDP) state they are inactive and become active by binding to guanosine triphosphate (GTP). The activation does not occur spontaneously but requires the assistance of guanosine nucleotide exchange factors (GEFs) that exchange the tightly bound GDP with GTP (Vetter and Wittinghofer, 2001). The inactivation is mediated by GTPase-activating proteins (GAPs), which stimulate the intrinsic GTP hydrolysis by accelerating its conversion to GDP and inorganic phosphate (Müller and Goody, 2018).

**Figure 1:**
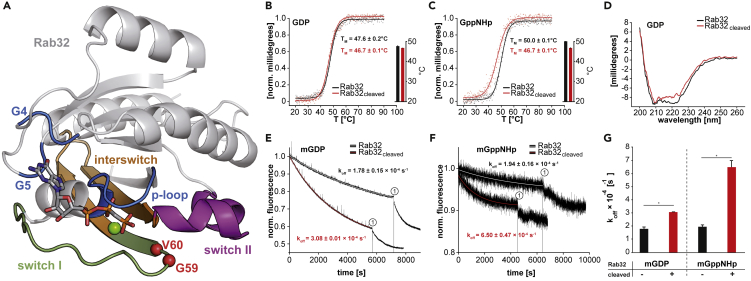
Biochemical and biophysical characterization of non-modified and cleaved Rab32 in both the activated and inactive states (A) The crystal structure of Rab32 (4CYM, Hesketh et al., 2014) in the active state is depicted with important structural regions highlighted: switch I (green), switch II (magenta), interswitch region (orange), and P loop, G4, and G5 (blue). GtgE-mediated cleavage occurs between G59 and V60 (red spheres). A non-hydrolyzable GTP analog (GppCp) is represented with sticks. The green sphere represents a magnesium cation. (B) GtgE-mediated proteolysis does not significantly impact the thermal stability of Rab32:GDP. Left: Normalized thermal unfolding curves of cleaved and uncleaved Rab32:GDP monitored via CD spectroscopy at 220 nm are plotted. The data are fitted with a Boltzmann function yielding the corresponding melting temperature (T_M_). Right: Comparison of T_M_ in both modification states using a bar graph representation. The data are presented as mean ± SEM (n = 2). (C) The thermal stability of Rab32:GppNHp is decreased upon GtgE-mediated proteolysis. Left: Normalized thermal unfolding curves of cleaved and uncleaved Rab32:GppNHp monitored via CD spectroscopy at 220 nm are plotted. The data are fitted with a Boltzmann function yielding the corresponding melting temperature (T_M_). Right: Comparison of T_M_ in both modification states using a bar graph representation. The data are presented as mean ± SEM (n = 2). (D) The CD spectra of uncleaved and cleaved Rab32:GDP. A comparison does not reveal any global structural differences. (E and F) mantGDP (E) and mantGppNHp (F) dissociation from Rab32 is elevated in the cleaved state. Mant fluorescence is shown as a function of time and decreases due to nucleotide release of mGDP (0.5 μM Rab32:mGDP, E) or mGppNHp (0.5 μM Rab32:mGppNHp) induced after the addition (at the time point 0) of a high concentration of non-fluorescent GDP or GppNHp (200 μM), respectively. The addition of 5 mM EDTA (1) maximizes the nucleotide release and leads to full dissociation of the mant-nucleotide. The fluorescence intensity and the time axis were normalized to the start of the reaction. (G) Quantification of nucleotide dissociation from uncleaved and cleaved Rab32 in both the activated and inactive states (data from E and F) are plotted in a bar graph. The data are presented as mean ± SEM (n = 3); ∗p < 0.05.

Among the small GTPases, the family of Rab proteins plays a crucial role in intracellular vesicular trafficking. Rab GTPases are reversibly membrane localized. The membrane localization is typically enabled by virtue of two geranylgeranyl lipids attached post-translationally to their structurally flexible C termini. Membrane delivery and recycling are directly coupled to the activation state of the Rab protein: In the inactive form, Rab proteins are cytosolic as they form a high-affinity complex with the GDP dissociation inhibitor (GDI), which shields the geranylgeranyl lipids in a hydrophobic binding pocket. In the active state, GDI cannot bind to the GTPase, leading to the liberation of the lipids, thereby mediating membrane localization. All interactions of GTPases with downstream effectors are mainly mediated via two conserved sequence regions called switch I and II, respectively (Figure 1A). The switch regions are structurally disordered in the inactive state and adopt a highly ordered structure in the active state due to the interactions of the GTP γ-phosphate with both regions (Vetter and Wittinghofer, 2001; Müller and Goody, 2018).

Many bacterial pathogens are taken up by human immune cells via phagocytosis and can survive inside the host. As human cells possess elaborate defense strategies against bacteria, the intruders have evolved strategies to manipulate signaling pathways of the host cell to their advantage. As Rab proteins control vesicular trafficking events (e.g., endosome fusion with lysosomes (Bucci et al., 2000)) that can lead to bacterial cleansing, they are particularly often targeted by bacterial enzymes or effectors (Müller et al., 2010; Spanò et al., 2011). A *Salmonella* infection caused by *S. enterica ssp. typhimurium* (*S.* typhimurium) can lead to the nonlethal but common salmonellosis (Crum-Cianflone, 2008). During infection, *S. typhimurium* secretes (in addition to other effectors) the cysteine protease GtgE via a type III secretion system into the cytosol of the host cell. GtgE targets the GTPase Rab32 subfamily, with Rab32 as the presumed main target (Spanò and Galán, 2012). This results in the proteolytic modification of switch I between G59 and V60 in Rab32, which was found to happen exclusively on inactive, GDP-bound Rab substrates (Spanò et al., 2011; Wachtel et al., 2018). Proteolysis may irreversibly and permanently inactivate Rab32 thereby preventing further interactions with regulating or interacting proteins. As a consequence, this would interfere with the delivery of antimicrobial factors and thereby support the *Salmonella* infection.

Rab32 has multiple functions in the cell. It is involved in the biogenesis of lysosome-related organelles, autophagy, mitochondrial dynamics and regulates phagosome maturation during bacterial invasion (Raposo et al., 2001; Alto et al., 2002; Holt et al., 2006; Wasmeier et al., 2006; Raposo and Marks, 2007; Benado et al., 2009; Bultema et al., 2012; Ao et al., 2014; Haile et al., 2017; Rybnicek et al., 2018; Hu et al., 2019). Moreover, in a recent study, Rab32 has been shown to facilitate the delivery of itaconate, a mitochondrial metabolite with antimicrobial activity, into the *Salmonella*-containing vacuoles (SCVs), thereby restricting the replication of *Salmonella* (Chen et al., 2020). To fulfill its functions, Rab32 must cycle between activity states. Thus, Rab32 is activated by the GEF BLOC-3 and can promote intracellular vesicular trafficking by binding to its effector protein VARP (Tamura et al., 2009; Gerondopoulos et al., 2012; Hesketh et al., 2014). Its inactivation can be mediated by its physiological GAP RUTBC1 or the bacterial effector SopD2 from *Salmonella* (Nottingham et al., 2011; Marubashi et al., 2016; Spanò et al., 2016).

The structural basis of the Rab32-GtgE interaction has been reported recently (Wachtel et al., 2018). The proteolytic cleavage of Rab32 by GtgE does not lead to protein unfolding, suggesting that the cleaved form of Rab32 (referred to as Rab32_cleaved_) may still be able to function in intracellular signaling (Wachtel et al., 2018). To investigate the molecular consequences of Rab32 cleavage, we conducted detailed biochemical, functional, and structural analyses. We compared the properties of intact Rab32 with Rab32_cleaved_. A combination of molecular dynamics (MD) simulations, nuclear magnetic resonance (NMR) experiments, and single-pair Förster resonance energy transfer (spFRET) analyses revealed an increase in the conformational flexibility of switch I and switch II. Additionally, Rab32_cleaved_ has an altered protein interaction profile, enabling GDI binding to inactive, cleaved Rab32 and fully impairing the Rab32-VARP interaction.

## Results

### Proteolytic modification of Rab32 affects its nucleotide binding

After GtgE-mediated cleavage of Rab32, the GTPase domain remains a stable and monomeric protein in solution (Wachtel et al., 2018). To dissect the functional consequences of Rab32 cleavage, we conducted detailed biochemical characterizations.

To elucidate if the proteolytic modification destabilizes the GTPase, we individually determined the protein melting temperature (T_M_) as a proxy for the stability of intact and cleaved Rab32 in the GTP-bound and GDP-bound states, respectively. Thermal unfolding monitored by the changes in the circular dichroism (CD) of secondary structure elements revealed that GtgE-mediated cleavage of Rab32:GDP causes a non-significant T_M_ decrease of 0.9°C (from 47.6°C to 46.7°C) (Figure 1B). Furthermore, the nucleotide exchange from GDP to GppNHp (a non-hydrolyzable GTP analog) results in an increased T_M_ value (50.0°C), which can be explained by the induced conformational order in the switch regions (Milburn et al., 1990). However, this stabilization effect is not reflected in the Rab32_cleaved_:GppNHp melting temperature (Figure 1C). Proteolysis of Rab32:GDP with subsequent nucleotide exchange does not affect the T_M_ (46.7°C) of either nucleotide states. Overall, the CD studies show that GppNHp has no stabilizing effect on cleaved Rab32 and its switch regions compared with the uncleaved state. Additionally, CD spectroscopy reveals that there are no notable substantial structural changes after GTPase proteolysis (Figure 1D).

Both switch regions contribute to the stabilization of the nucleotide in the nucleotide-binding pocket of small GTPases (Pai et al., 1989; Milburn et al., 1990; Scheidig et al., 1999). Thus, the nucleotide dissociation properties may be used to investigate the impact of GtgE-mediated cleavage on the stability of the switch regions. To investigate the nucleotide dissociation rates, Rab32 was loaded *in vitro* with modified GDP or GppNHp bearing the fluorescent 2'/3′-O-(N-Methyl-anthraniloyl) (mant) moiety attached to the ribose (mGDP or mGppNHp). The nucleotide dissociation was monitored by the decrease in mant fluorescence intensity as a function of time after the addition of an excess amount of non-fluorescent counterparts (GDP or GppNHp). The nucleotide dissociation rate (k_off_) of the proteolytically cleaved Rab32 increased regardless of the nucleotide state. Rab32:mGDP shows a k_off_ value of (1.78 ± 0.15) × 10^−4^ s^−1^ for non-modified and (3.08 ± 0.01) × 10^−4^ s^−1^ for the cleaved state. On the other hand, Rab32:mGppNHp has a k_off_ value of (1.94 ± 0.16) × 10^−4^ s^−1^ for uncleaved and (6.50 ± 0.47) × 10^−4^ s^−1^ for the cleaved state (Figures 1E and 1F). Thus, the nucleotide dissociation rate is almost doubled for mGDP or tripled for mGppNHp in cleaved Rab32 (Figure 1G). As GtgE proteolytically cleaves the switch I region, which is involved in nucleotide binding and magnesium ion coordination, it is not surprising that the cleavage leads to an increased nucleotide dissociation rate caused by destabilization of the switch I.

### Proteolysis reduces binding of Rab32 to the VARP-ANK1 domain

**Figure 2:**
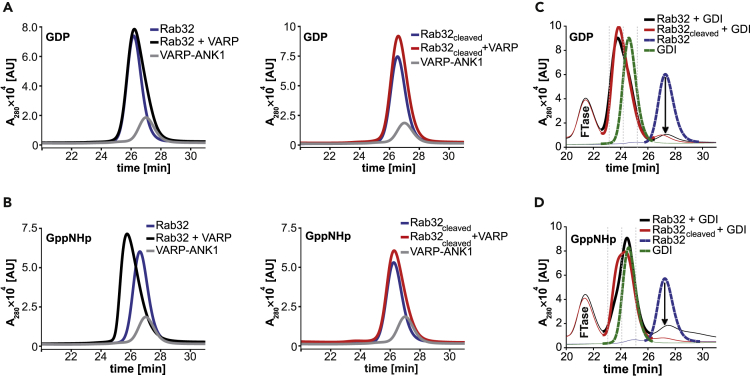
Binding of Rab32 with its physiological interaction partners is selectively impaired by the proteolytic modification To investigate the interaction of Rab32 with other proteins, aSEC measurements were preformed where the intensity at 280 nm was monitored and the resulting peaks were deconvolved into the individual species. (A) Left: aSEC measurements of Rab32:GDP in the presence of VARP. VARP-ANK1 does not bind Rab32:GDP *in vitro*. Rab32 (50 μM) was preparatively loaded with GDP (98%) and equilibrated for complex formation with 50 μM VARP-ANK1 for 1 h at 15°C. Subsequently, 50 μL was chromatographically separated via aSEC. The individual runs of single proteins serve as a reference. Right: aSEC measurements of cleaved Rab32:GDP in the presence of VARP. Cleaved Rab32:GDP also does not form a complex with VARP-ANK1. (B) Left: Complex formation between active Rab32 and VARP-ANK1 investigated using aSEC. The analysis corresponds to that used in (A) starting with Rab32:GppNHp (90% loaded). Here, clear complex formation is observed. Right: aSEC measurement of the interaction between cleaved Rab32:GppNHp and VARP-ANK1. Cleavage of Rab32 impairs the complex formation between Rab32 and VARP-ANK1. (C) aSEC measurements of the interaction of Rab32:GDP with GDI in the uncleaved and cleaved states. Inactive Rab32 (100% loaded with GDP) binds GDI regardless of the state of its proteolytic modification. (D) aSEC measurements of the interaction of Rab32:GppNHp with GDI in the uncleaved and cleaved states. Rab32_cleaved_:GppNHp can more efficiently form a complex with GDI when compared with the uncleaved Rab32. This can be clearly seen in the reduction of the Rab32 only peak for Rab32_cleaved_:GppNHp when compared with Rab32:GppNHp (arrow); 30 μM B12 was used as internal standard for each aSEC run.

As proteolysis of Rab32 may affect binding to its effectors, we investigated the interaction between Rab32 and its physiological interaction partners, the ANK1-domain of VARP and GDI. VARP is an effector of Rab32 that specifically binds to the uncleaved active GTPase through its ANK1-domain (Tamura et al., 2009). Assuming the exchange from Rab_cleaved_:GDP to Rab_cleaved_:GTP is not impaired by the proteolytic modification, the interaction with VARP might still be possible. In case the nucleotide exchange is prevented by the cleavage, it would render Rab32_cleaved_:GDP a dead end for the downstream signaling with VARP. However, Rab32_cleaved_:GDP might still be able to bind to GDI. To test both scenarios, we prepared uncleaved and cleaved Rab32 in each activation state *in vitro* and tested their binding capability with VARP and GDI, respectively, via analytical size exclusion chromatography (aSEC) (Figure 2).

As expected, neither Rab32:GDP nor its proteolytically modified counterpart form a complex with the VARP-ANK1, whereas Rab32:GppNHp forms a stable complex with the ANK1-domain (Figures 2A and 2B) (Hesketh et al., 2014). However, there is no binding observed between Rab32_cleaved_:GppNHp and VARP-ANK1 (Figure 2B, left). Thus, the cleavage of Rab32 by GtgE impairs its binding to VARP-ANK1 *in vitro*.

GDI specifically interacts with the inactive state of prenylated Rab GTPases (Sasaki et al., 1990; Wu et al., 2007). The lipid anchor on the C terminus of Rab GTPases is crucial for the interaction with GDI (Wu et al., 2007). To this end, we produced Rab32 bearing the CVIM-sequence on its C terminus for farnesylation (Joberty et al., 1993). Subsequently, it was proteolyzed by catalytic amounts of GtgE, loaded with the desired nucleotide and farnesylated, as described in Transparent methods. Farnesylation was confirmed by mass spectrometry of the intact proteins before and after lipidation (Figure S1). Surprisingly, farnesylated Rab32_cleaved_:GDP appears to form a complex with GDI, indicated by the reduced elution time of the complex as well as the decreased Rab32 peak (Figures 2C and S2). In contrast to the well-known binding preference of GDI for GDP-bound Rab proteins, the chromatographic data suggest that GDI is also able to form a complex with Rab32_cleaved_:GppNHp (Figure 2D). Peak decomposition indicates distinct complex formation between GDI and Rab32 in the cases of Rab32:GDP, Rab32_cleaved_:GDP, and Rab32_cleaved_:GppNHp (Figure S3). Of note, uncleaved prenylated Rab32:GppNHp seems to form a complex with GDI to some extent (Figure S3). This could be explained by not 100% GppNHp-loaded Rab32 (85% GppNHp). Thus, the resulting complex between Rab32:GppNHp and GDI could be formed by GDP-loaded species present in the sample.

In conclusion, the proteolytic modification of Rab32 results in the disruption of its interaction with VARP-ANK1. Furthermore, the proteolytic cleavage has no effect on the interactions with GDI in the case of Rab32_cleaved_:GDP; however, in the case of Rab32_cleaved_:GppNHp, the binding is increased compared with its uncleaved state.

### GtgE-mediated cleavage destabilizes switch I and indirectly impacts switch II of Rab32

**Figure 3:**
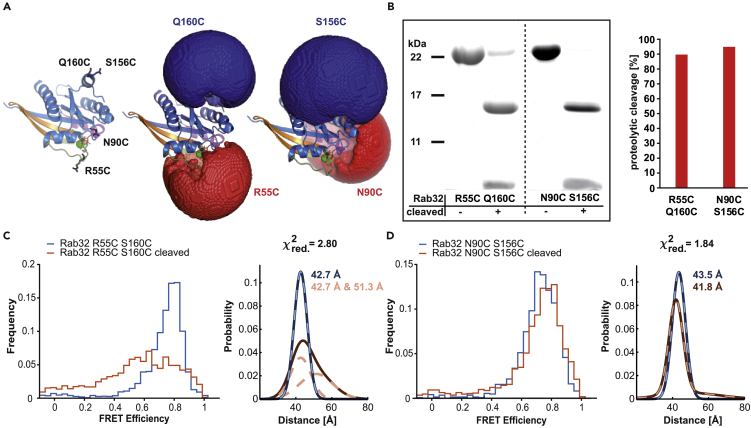
Single-pair FRET reveals changes in the conformation of Rab32 upon proteolytic modification (A) Position of Cys mutations within the Rab32 for covalent fluorophore linkage. Left: Ribbon structure representation of Rab32 where the selected amino acid positions for protein labeling with FRET pairs are shown as sticks. Middle and right: Visualization of the accessible volume calculations for Rab32_R55C Q160C_ and Rab32_N90C S156C_ double mutants, respectively. The Rab32 structure used for the current representation is deposited in the PDB under the ID 6FF8 (McGrath et al., 2019). (B) Quantification of cleavage efficiency for the spFRET Rab32 mutants. Left: Rab32:GDP mutants were proteolytically cleaved and run on an SDS-PAGE gel. Right: Densitometric quantification of the gel bands for modification completion in both double mutants plotted in a bar graph. (C) SpFRET histograms for the uncleaved and cleaved Rab32_R55C Q160C_ mutant revealing a change in distance between fluorophores upon cleavage. The distances calculated using probability distribution analysis approach are indicated. Orange dashed lines show the decomposed peak after proteolysis with two equally populated species. (D) SpFRET histograms for the uncleaved and cleaved Rab32_N90C S156C_ mutant. Cleavage of switch I leads to minor distance differences in the switch II region of the protein. All calculated distances represent the separation of the fluorophores, which are attached to the protein via flexible linkers.

Next, we investigated the impact of the proteolytic modification in switch I on the conformations of both switch regions of Rab32. To this end, we applied spFRET, which allows the determination of conformational changes within disordered protein regions by detecting distance variations on individual molecules (LeBlanc et al., 2018). Strategically positioned pairs of Cys substitutions within Rab32 served as fluorophore labeling sites. As Rab32 is a small protein, we strived to maximize the distances between the labeling positions on the static portion of the protein (Q160C or S156C) and a potentially dynamic counterpart in either the switch I (R55C) or switch II (N90C) region of the protein (Figure S4). Subsequently, spFRET-suitable fluorophores (Alexa 488-maleimide, Alexa 647-maleimide) bearing a maleimide moiety were covalently coupled to Cys-containing Rab32_R55C/Q160C_ or Rab32_N90C/S156C_ constructs (Figure 3A, left). Intrinsic Cys residues (C145, C162) were mutated to Ser to avoid off-target labeling. The coupled dyes contain flexible linkers not only allowing free rotation but also increasing the volume in which the dye molecule can be located (Table S1). To visualize the spatial distribution of the possible locations of the fluorescent dye, we performed accessible volume calculations that estimate the possible locations of the fluorophore based on its size and the size and length of the linker (Kalinin et al., 2012). Accessible volume calculations for both Rab32 double mutants are presented in Figure 3A. The main goal for the FRET experiments is to detect conformational changes upon cleavage between residues 59 and 60. For this purpose, we also generated pure cleaved Rab32 variants ready for fluorophore labeling and subsequent spFRET measurements (Figure 3B). The proteolytic modification of Rab32_R55C/Q160C_ labeled in switch I generates two species with equal contributions of 50% after peak decomposition (Figure 3C). The distance of the first species corresponds to uncleaved Rab32, whereas there is an increase in distance of the second species of 8.6 Å between the fluorophores after cleavage (Figure 3C). In the case of the Rab32_N90C/S156C_ labeled in switch II, only minor changes are observed. The cleavage reduces the distance between the fluorophores by ∼2 Å (Figure 3D).

In summary, spFRET data indicate a strong destabilization of the switch I region as well as an indirect minor influence on switch II of Rab32 upon the GtgE-mediated proteolysis.

### Rab32_cleaved_:GppNHp exhibits structural similarities with its GDP state

**Figure 4:**
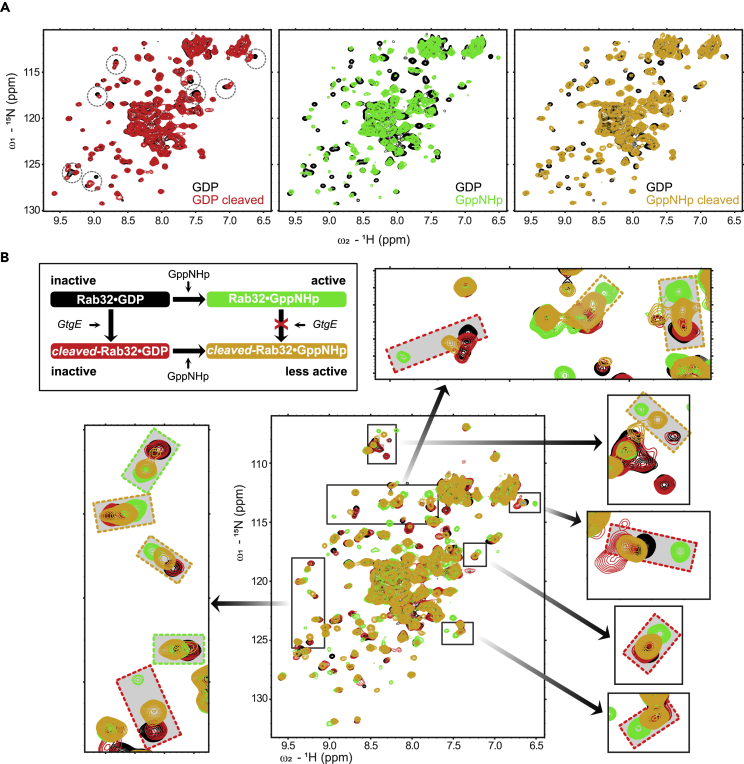
NMR analysis of structural effects of proteolysis and nucleotide binding on Rab32 (A) Superposition of ^1^H, ^15^N NMR correlation spectra of ^15^N-labeled Rab32 with GDP (black) or GppNHp (green) and their cleaved states (red and orange, respectively) by GtgE. Note that the cleaved Rab32:GppNHp (orange) has been produced by proteolysis of Rab32:GDP followed by addition of GppNHp. Specific spectral changes of the GDP state upon cleavage are highlighted with dashed circles. (B) Summary of the states of Rab32 used for NMR analysis. Spectral overlays of the four states indicate that the NMR signals of the cleaved GppNHp-bound state (orange) generally are more similar to the inactive state (black, red), where the cleaved GppNHp-bound state signals (orange) appear close to the inactive state (red boxes) or intermediate (orange boxes) between the GppNHp-bound active (green) and GDP-bound (black) states. The NMR signals with the cleaved GppNHp-bound state (orange) that correlate with the active state (green) are shown in green boxes.

For a better understanding of the structural consequences of GtgE proteolysis in Rab32, NMR studies were conducted (Figures 4 and S5). For this purpose, we recorded and compared ^1^H, ^15^N heteronuclear single quantum correlation (HSQC) NMR spectra of the four states (indicated in the schematics in Figure 4B), identifying the effects of GtgE-mediated proteolysis in the presence of different nucleotides (GDP, GppNHp). First, replacement of GDP to GppNHp (non-hydrolyzable GTP analog) in Rab32 shows large spectral differences, where most signals are shifted or broadened in the GppNHp state, that represents the GTP-bound form (Figure 4A, black versus green). This suggests that GTP-induced transition to the active state is not limited to local changes in the switch region, but rather results in a more global conformational change in Rab32. Next, we investigated GtgE-mediated cleavage of Rab32 in the presence of GDP or GppNHp. Upon GtgE-mediated cleavage of Rab32:GDP, a small set of NMR signals is clearly shifted or broadened (Figure 4A, black versus red), likely corresponding to the residues surrounding the cleavage site in the switch region. In contrast, virtually no spectral changes are observed for Rab32:GppNHp upon adding a catalytic amount of GtgE (Figure S5). This indicates that the nucleotide exchange to GppNHp was complete and confirms the preference for GtgE to interact with GDP-bound Rab32 (Wachtel et al., 2018). Last, we tested the effect of GppNHp-binding on the cleaved Rab32:GDP (Rab32_cleaved_:GppNHp). Spectral changes indicate nucleotide exchange from GDP to GppNHp for cleaved Rab32. However, most of the signals do not reach the fully active GppNHp-bound state but rather appear between the two states (Figure 4B, orange box) and are closer to the GDP-bound state (Figure 4B, red box), whereas a few signals of Rab32_cleaved_:GppNHp can be linked to the Rab32:GppNHp state (Figure 4B, green box). In short, the NMR spectral comparisons indicate that (1) GppNHp binding triggers conformational changes beyond the switch region and protects the switch region from proteolysis and (2) GtgE-mediated proteolysis results in local changes in the switch region, which subsequently lock Rab32 in an inactive-like conformational state despite its nucleotide state.

### GtgE-mediated cleavage destabilizes the switch regions and disrupts the interswitch region of Rab32

Additionally, we performed MD simulations on cleaved and uncleaved Rab32 in active and non-active conformations. MD simulations are a complementary approach to gain further insights regarding stability and the timescale of the dynamic changes in both switch regions upon the proteolytic modification. Moreover, MD simulations can provide additional structural reasons for the impaired binding between the VARP-ANK1 domain and Rab32_cleaved_:GppNHp.

**Figure 5:**
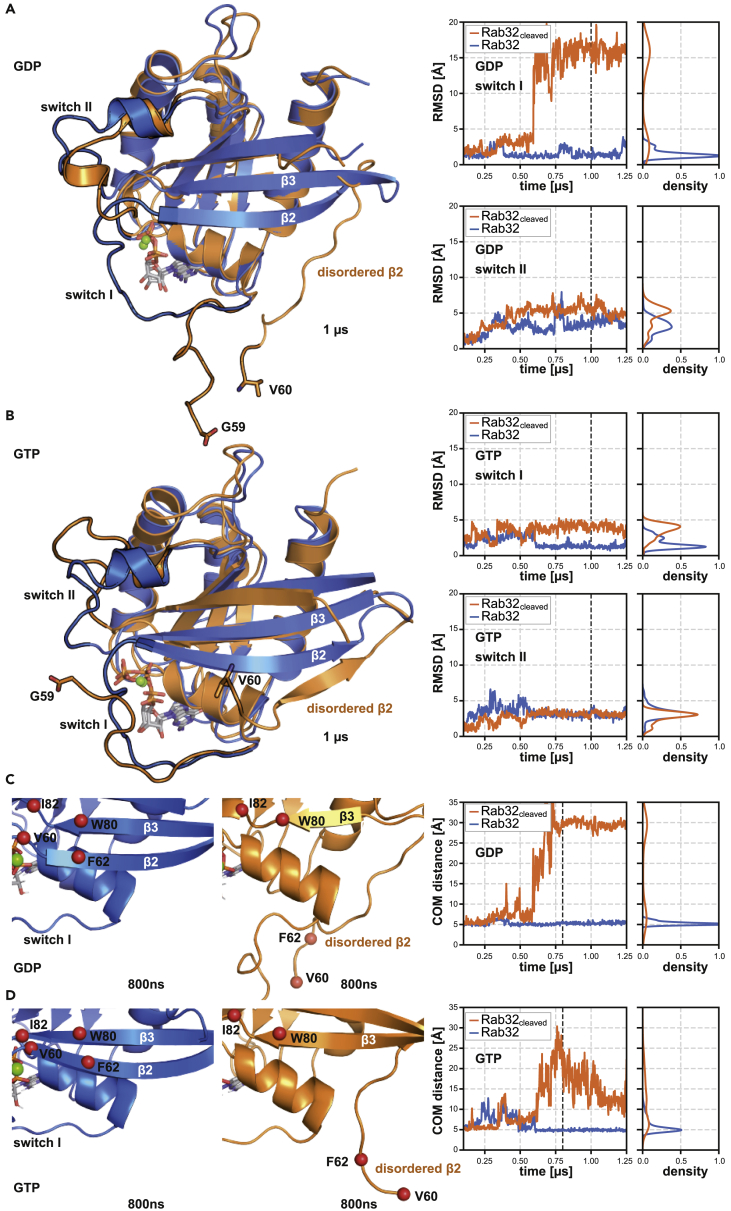
Cleavage-induced flexibility in the switch I promotes unfolding of the β2-strand in the interswitch region of Rab32 (revealed by molecular dynamics simulations) (A) Left: Superimposed MD simulated structures of Rab32:GDP (blue) and Rab32_cleaved_:GDP (orange, the disordered β2-strand is indicated) after 1 μs of MD simulations. The switch regions are highlighted with black outline. Right: RMSD versus simulation time for switch I and II regions of Rab32:GDP and Rab32_cleaved_:GDP. The black dashed lines indicate the sampling time of the corresponding snapshots shown on the right. (B) Left: Superimposed MD simulated structures of Rab32:GTP (blue) and Rab32_cleaved_:GTP (orange) after 1 μs. Right: Same as in (A) for the Rab32:GTP and Rab32_cleaved_:GTP. (C) Left and middle: The interswitch region of Rab32:GDP (blue) and Rab32_cleaved_:GDP (orange) presents the β2 strand at 800 ns of the MD simulation with a large change of the COM (center-of-mass) distances of V60 & F62 and W80 & I82 in the cleaved versus uncleaved states. Red spheres indicate the C_α_-atoms of the amino acids V60, F62, W80, and I82. Right: V60 & F62 - W80 & I82 COM distances versus simulation time. The point of 800 ns, indicated by the black dashed line, highlights the time point of the snapshots shown in the left and middle panels. (D) Same as in (C) but for the versus Rab32:GTP case.

Simulations were started from the active GTPase conformation with either a bound GDP or GTP and either an intact protein chain or with cleavage between G59 and V60 (see Transparent methods). In the presence of GDP, proteolytic modification leads to a drastically increased root-mean-square deviation (RMSD) of both switch regions in the inactive state of Rab32 relative to the non-modified state, indicating destabilization (Figure 5A). The distributions of the RMSD values demonstrate sampling of conformations for the switch I and switch II regions that deviate from the start structures by ∼16 Å and ∼5 Å, respectively (Figures 5A and 5B right). The following simulations in the presence of bound GTP revealed that GTP binding is not sufficient to maintain the stability and structure of switch I in the cleaved form (Figure 5B, left). Similarly, the RMSD plot in the cleaved state indicates that switch I has higher flexibility compared with its uncleaved state, reaching an RMSD of around 4 Å (Figure 5B, right). However, on the timescale of the simulations, no differences in the flexibility of switch II in case of the cleaved versus uncleaved Rab32 with bound GTP were observed (Figure 5B, right). The simulations indicate that proteolysis of Rab32 disrupts and unfolds the interswitch region of Rab32 independently of its nucleotide-bound state (Figures 5C and 5D, left and middle). This disruption appeared after 500 ns of simulation time and led to the loss of structure of the β2 strand. Moreover, the antiparallel β-sheet strands β2 and β3 drift apart during the simulation (Figures 5C and 5D middle). This is reflected in an increase of the center-of-mass (COM) distance of two residue pairs in the two strands (W80&I82 and V60&F62) observed for both GDP- and GTP-bound cleaved Rab32 in the course of the simulations (see Figures 5C and 5D, right). The local increase in flexibility of the switch regions and unfolding of the interswitch region in cleaved Rab32 observed in the MD simulations is consistent with the spFRET and NMR results, whereas the dissociation of the β2 strand appears not to be reflected in the NMR spectra. Hence, the MD simulations indicate qualitatively the enhanced mobility of the switch and interswitch regions in Rab32 due to cleavage in good agreement with the spFRET and NMR results, but appear to overestimate the effect of cleavage on the β2 strand mobility. Nevertheless, the observed destabilization of the switch regions and unfolded interswitch region of cleaved Rab32 during the simulations explains the reduced interaction of cleaved Rab32 with VARP-ANK1. Both switches and the interswitch region are at the interface in the complex with VARP-ANK1. Consequently, consistent with spFRET data, MD simulations show that proteolysis significantly destabilizes switch I as well as slightly impacts the conformation of switch II. Furthermore, the interswitch region of Rab32 is dramatically disordered independently of its nucleotide state upon proteolysis. Both switches and the interswitch region are at the interface in the complex with VARP-ANK1 domain explaining the reduced interaction of cleaved Rab32 with VARP-ANK1.

## Discussion

In this study, we present structural and biochemical investigations on the consequences of GtgE-mediated cleavage of inactive Rab32. Our findings show that the proteolytic modification of switch I does not affect the global structural stability of the protein but leads to local changes of the structure and destabilization of switch I and switch II. Moreover, the regioselective cleavage of switch I disrupts the interswitch region of Rab32 leading to unfolding of the β-sheet structure of the β2 strand. Moreover, Rab32_cleaved_:GppNHp is not able to interact with its effector VARP-ANK1. In contrast, proteolysis does not impair the interaction between Rab32:GDP and GDI. Furthermore, Rab32_cleaved_:GppNHp but not Rab32:GppNHp is able to bind to GDI.

**Figure 6:**
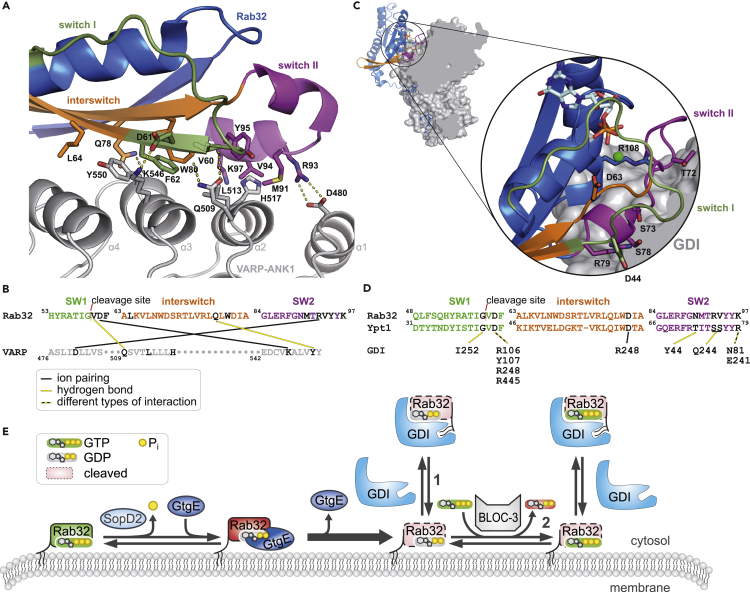
Models of the molecular basis for binding effects of cleaved Rab32 with its interaction partners (A) Structural representation of Rab32 (colored) and VARP-ANK1 (gray) with important interaction residues presented as sticks (PDB: 4CYM, Hesketh et al., 2014). (B) The sequence of the switch I, switch II, and interswitch region of Rab32 with corresponding interacting amino acids from VARP-ANK1 depicted in black. Salt bridges between amino acids are indicated by black-yellow lines. (C) A structural representation of Ypt1 (yeast GTPase) and GDI showing the interaction surface and important interaction residues of switch I, switch II, the interswitch region, and the C-terminal region of Ypt1 with hydrophobic moiety (PDB: 2BCG, Pylypenko et al., 2006). Structural representations of the proteins were prepared using PyMol. (D) Sequence comparison of the GDI-interacting regions of Ypt1 and Rab32 with crucial amino acids for the GDI binding depicted in black. Amino acids contributing to the interaction between the two protein structures are shown in black. (E) Model of the hypothetical life cycle of Rab32 during the *Salmonella* infection process with possible routes for proteolyzed Rab32. Once the intrinsic hydrolysis of GTP to GDP occurs in Rab32, which is accelerated by SopD2 from *Salmonella*, it can be processed by GtgE protease and take one of two possible pathways, indicated with arrows 1 or 2, respectively.

Amino acid residues from switch I (V60, D61, and F62), switch II (M91, V94, R93, Y95, and K97) and the interswitch region (D61, L64, and W80) are crucial to establishing an interaction surface between Rab32 and VARP-ANK1 (Figures 6A and 6B) (Hesketh et al., 2014). Interestingly, the Met and Arg residues from switch II, which correspond to positions 91 and 93 in Rab32, respectively, are conserved only in the Rab32 subfamily and are vital for the interaction with the ankyrin repeats (Hesketh et al., 2014; Purlyte et al., 2018). The importance of the interswitch region of small GTPases for the interaction with their effectors has been reported previously (Zhu et al., 2004; Eathiraj et al., 2005, 2006; Wu et al., 2005; Jagoe et al., 2006). Likely, the gain in structural flexibility of the switch regions of cleaved Rab32 accompanied by interswitch unfolding indicated by spFRET data and MD simulations lead to a disturbance of the interaction between Rab32_cleaved_:GppNHp and its effector. Additionally, proteolytic modification of Rab32 forces β2 and β3 strands to drift apart, as indicated by the increased COM distances between these two β strands in the MD simulations. This, in turn, displaces all residues of the β2 strand involved in the interaction with VARP-ANK1 and, in this fashion, contributes to break down of the Rab32:VARP-ANK1 interaction. VARP participates in trafficking of melanogenic enzymes and has been suggested to control the transport in the endosome-to-cell surface route by regulating the activity of retromer (Tamura et al., 2009, 2011; Hesketh et al., 2014; Marubashi et al., 2016). However, the role of Rab32 as a binding partner of VARP is not entirely understood (Hesketh et al., 2014). The destruction of the interaction between Rab32 and VARP may lead to a missorting and degradation of melanogenic enzymes or disruption of trafficking out of the endosome in the cell periphery (Tamura et al., 2009, 2011; Bultema et al., 2012; Hesketh et al., 2014; Marubashi et al., 2016).

Moreover, a recent study has shown that proteolytic modification of the switch I in KRas by the Ras- and Rap1-specific protease (RRSP) from *Vibrio vulnificus*, analogous to the activity of GtgE in Rab32, impairs the binding to its interaction partner RAF (Biancucci et al., 2018). In contrast to GtgE, RRSP cleaves switch I of KRas in its middle and not near its C-terminal end. Analogous to GtgE-mediated proteolysis, cleavage by RRSP impacts the β2 strand of KRas (Biancucci et al., 2018). However, Rab32 and KRas differ in the nucleotide binding properties after proteolytic modification: Cleaved Rab32 but not KRas shows elevated GDP/GTP dissociation rates. Stable nucleotide binding in KRas is assumed to be ensured by F28 binding to the guanine moiety, but the identically positioned F50 in Rab32 is not sufficient for nucleotide stabilization after GtgE-mediated proteolysis (Biancucci et al., 2018). Additionally, the results of our spFRET experiments and MD simulations show that switch I gains structural flexibility after proteolysis and thus likely does not contribute to the stabilization of the nucleotide in its binding pocket. The existence of two species of cleaved Rab32_R55C/Q160C_, as demonstrated by two different distances between the fluorophores from the spFRET measurements, could be explained by dynamic behavior of the proteolyzed switch I region. Thus, switch I of Rab32_cleaved_ is not displaced from the protein core permanently, but attaches and detaches dynamically. Therefore, the position of the cleavage site within switch I plays an important role in its conformational stability and the nucleotide-binding ability.

Changes in the Rab32 structure caused by proteolysis are not enough to impair the interaction with GDI. Comparing Rab32 with Ypt1 in complex with GDI, G59, and D61 from switch I; N90, R93, V94, and K97 from switch II; and only D81 from the interswitch region contributes to the Rab32:GDI complex formation (Figures 6C and 6D) (Pylypenko et al., 2006). As the proteolytic modification has an immense influence on the localization of the interswitch region and switch I, they are apparently not vital for the interaction with GDI. Therefore, the Rab32 binding to GDI is presumably determined by the structural organization of switch II. Noteworthy, bulky post-translational modifications on switch II (such as AMPylation, enzymatic transfer of AMP moiety to a target molecule) of Rab1b impair the interaction with GDI (Goody et al., 2012; Oesterlin et al., 2012; Levin et al., 2016). Nonetheless, the indirect minor impact of GtgE-mediated cleavage on the switch II has a big consequence for the interaction of Rab32 with GDI resulting in the ability to bind to Rab32_cleaved_:GDP and Rab32_cleaved_:GTP. Recently, it has been shown that bacteria can lock Rab1b in the active state using AMPylation (Barthelmes et al., 2020). Consequently, *Salmonella* may use proteolysis to force Rab32 into an inactive-like conformation as demonstrated here by NMR. This may explain the ability of Rab32_cleaved_ to interact with GDI in the GDP and GTP states.

Once proteolyzed, cellular Rab32-signalling could hypothetically develop in two ways: (1) immediate interaction with GDI and withdrawal from the membrane or (2) GEF-mediated nucleotide exchange to GTP followed again by GDI interaction and withdrawal from the membrane (Figure 6E). The first route seems to be adequate, whereas the second one is questionable, as Rab32 must be first activated by its GEF BLOC-3. Whether cleaved Rab32 can interact with BLOC-3 has yet to be elucidated. BLOC-3 belongs to heterodimeric RabGEF complexes and exhibits similarities with the Mon1-Ccz1 complex. Therefore, BLOC-3 probably utilizes the same mechanism for the nucleotide exchange as the Mon1-Ccz1 complex does (Nordmann et al., 2010; Kiontke et al., 2017). Furthermore, Rab32 possesses an R55 matching with the K38 of Ypt7, which is crucial for its interaction with Mon1-Ccz1 (Kiontke et al., 2017). As our results show that cleaved switch I has higher flexibility and is dislocated, it would not be surprising if proteolytically modified Rab32 cannot be activated by BLOC-3. Therefore, GtgE-mediated proteolysis may possibly ensure the complete removal of Rab32 by GDI from the SCV membrane enabling the survival of the bacteria within the host cell (Spanò and Galán, 2012; Hu et al., 2019). Interestingly, HeLa cells have a similar copy number of Rab32 and GDI (Itzhak et al., 2016). Thus, an additional effect of Rab32_cleaved_:GDP and Rab32_cleaved_:GppNHp may be a GDI depletion from the host cell securing the higher membrane localization of other Rab GTPases. Furthermore, the proteolytic constitutive deactivation of Rab32 may explain the impairment of itaconate delivery into the SCVs in a Rab32-BLOC3-dependent manner (Chen et al., 2020).

GtgE targets also the Rab32 homolog Rab29 and proteolyzes its switch I between G41 and V42, which is comparable to the cleavage site in Rab32 (Spanò et al., 2011). Moreover, Rab29 interacts with the Armadillo domain of leucine-reach repeat kinase 2 (LRRK2) (McGrath et al., 2019). The result of this interaction is LRRK2 recruitment and activation on the Golgi (Purlyte et al., 2018). Noteworthy is also the activation of the NLRC4 inflammasome by active LRRK2 during *Salmonella* infection (Liu et al., 2017). Therefore, it would be of great interest to investigate whether Rab29 proteolysis by GtgE may impair the Rab29-mediated LRRK2 recruitment and activation on the Golgi, and reduce the activation of NLRC4 inflammasome (Liu et al., 2017; Purlyte et al., 2018). It would also be beneficial to understand whether the kinase activity of LRRK2 during the infection is associated with Rab32 and plays a pivotal role in the defense mechanism of the host against *Salmonella* (Gardet et al., 2010; Liu et al., 2017).

In this study, we demonstrate that the high flexibility and dislocation of switch I, the alteration of the interswitch region, and locking of Rab32 in a GDP-like state by GtgE-mediated cleavage are responsible for impairing the interaction between VARP-ANK1 and Rab32 as well as for binding of GDI to Rab32_cleaved_:GDP or Rab32_cleaved_:GppNHp. These findings expand our understanding about the consequences of GtgE-mediated proteolysis on Rab32, which facilitates *Salmonella* infection. Withal, we provide possible further downstream effects of proteolytic modification of Rab32 and deepen the knowledge about mechanisms of *Salmonella* infection.

### Limitations of the study

Results of this study provide a comprehensive insight into the consequences of proteolytic modification of Rab32. However, the ability of cleaved Rab32 to interact with its physiological GEF BLOC-3 and, thereby, be activated remains speculative. In addition, the physiological consequences of the observed *in vitro* effects of Rab32 cleavage will require further elucidation.

### Resource availability

#### Lead contact

Further information and requests for resources and reagents should be directed to and will be fulfilled by Aymelt Itzen (a.itzen@uke.de).

#### Materials availability

All unique materials generated in this study are available from the Lead Contact without restriction.

#### Data and code availability

This study did not generate or analyze datasets or code.

## Methods

All methods can be found in the accompanying Transparent methods supplemental file.

## Supplemental information

Document S1. Transparent methods, Figures S1–S5, and Table S1
