# Supplementary material for: Proteolysis of Rab32 by Salmonella GtgE induces an inactive GTPase conformation
Source: iScience. 2020 Dec 15;24(1):101940. doi: 10.1016/j.isci.2020.101940 (PMC7779776; doi:10.1016/j.isci.2020.101940)
Supplement: Document S1. Transparent methods, Figures S1–S5, and Table S1 [file mmc1.pdf]

iScience, Volume *24*

Supplemental Information

Proteolysis of Rab32
by *Salmonella* GtgE induces
an inactive GTPase conformation

Sergey Savitskiy, Rudolf Wachtel, Danial Pourjafar-Dehkordi, Hyun-Seo Kang, Vanessa Trauschke, Don C. Lamb, Michael Sattler, Martin Zacharias, and Aymelt Itzen

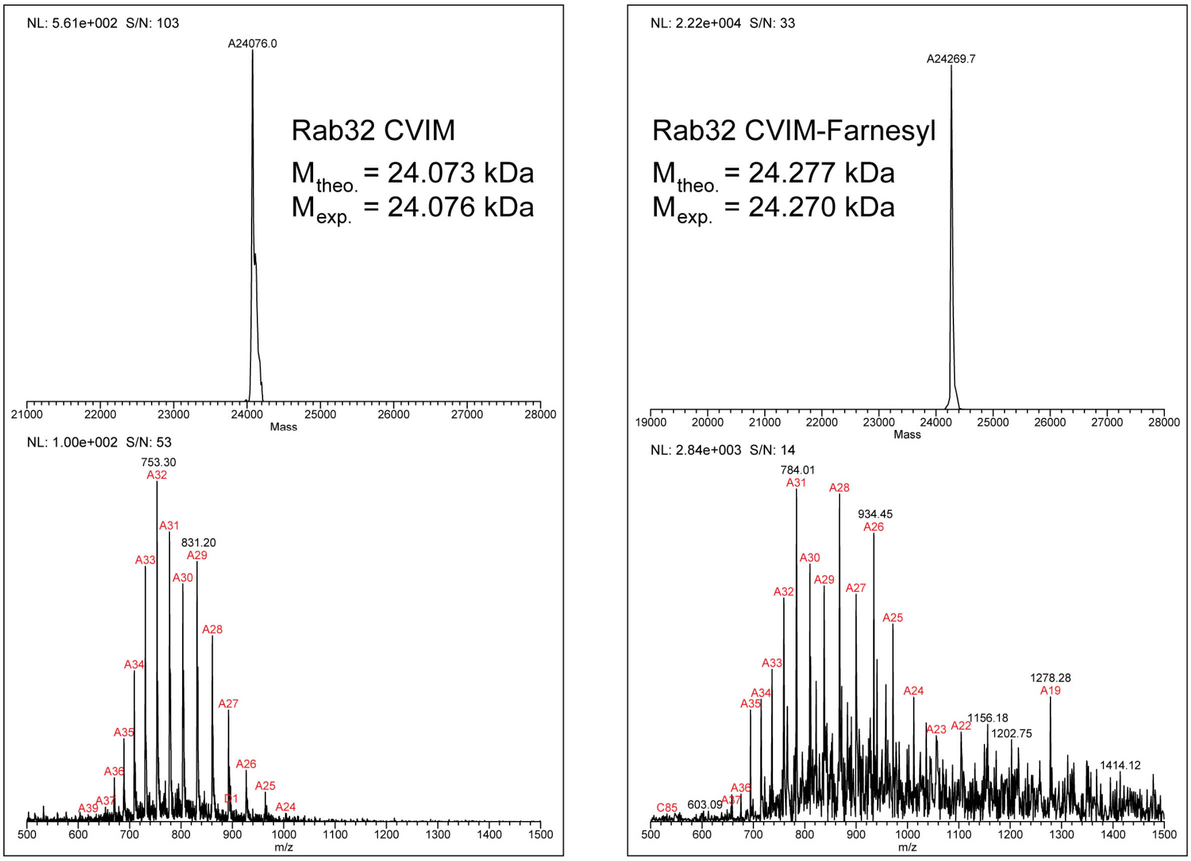

Figure S1: Mass spectrometry data of Rab32 CVIM farnesylation. Related to Figure 2C and 2D. Left: unmodified Rab32 bearing the CVIM-sequence. Right: farnesylated Rab32 CVIM. Both masses were determined with an offset of 3 Da ($M_{exp.}$) compared to the calculated, theoretical mass ($M_{theo.}$) The measured difference in mass ($\Delta M_{exp.}$ = 194 Da) does not correspond fully to the theoretical Mass difference of an attached farnesyl moiety ($\Delta M_{theo.}$ = 204 Da). The difference of 10 Da is due to the mass resolution limitations of the instrument and m/z-data quality of the farnesylated Rab32 which showed significantly less ionization upon modification. The deconvoluted mass spectra (top) and the m/z spectra (bottom) are demonstrated.

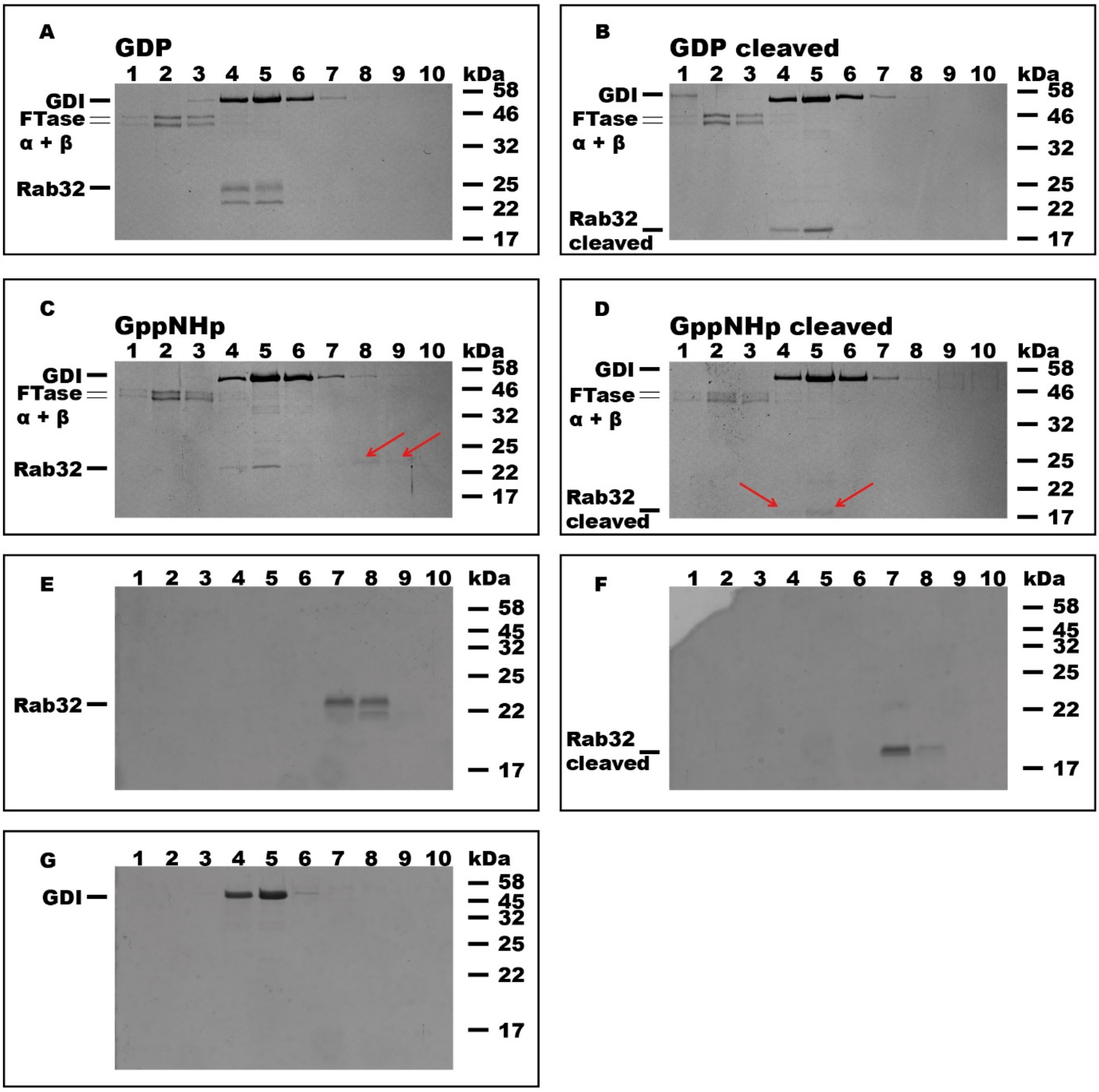

Figure S2: Fraction analysis of the Rab32:GDI complex formation on an aSEC. Related to Figure 2C and 2D. Coomassie stained SDS-PAGE of fractions from aSEC separations containing farnesylated Rab32 CVIM in the presence of GDI and FTase (compare Fig. 2C, 2D). **A** Rab32:GDP. **B** $Rab32_{cleaved}$:GDP. **C** Rab32:GppNHp. **D** $Rab32_{cleaved}$:GppNHp. Slightly visible bands are highlighted with red arrows. All Rab32 preparations (A-D) are forming a non-covalent complex with GDI indicated by the coelution in fractions 4 and 5. Only Rab32:GppNHp (C) shows a partial complex formation indicated by free Rab32 in fractions 8 and 9. **E-G** Control runs of purified proteins (**E**) Rab32, (**F**) $Rab32_{cleaved}$ and (**G**) GDI on an aSEC.

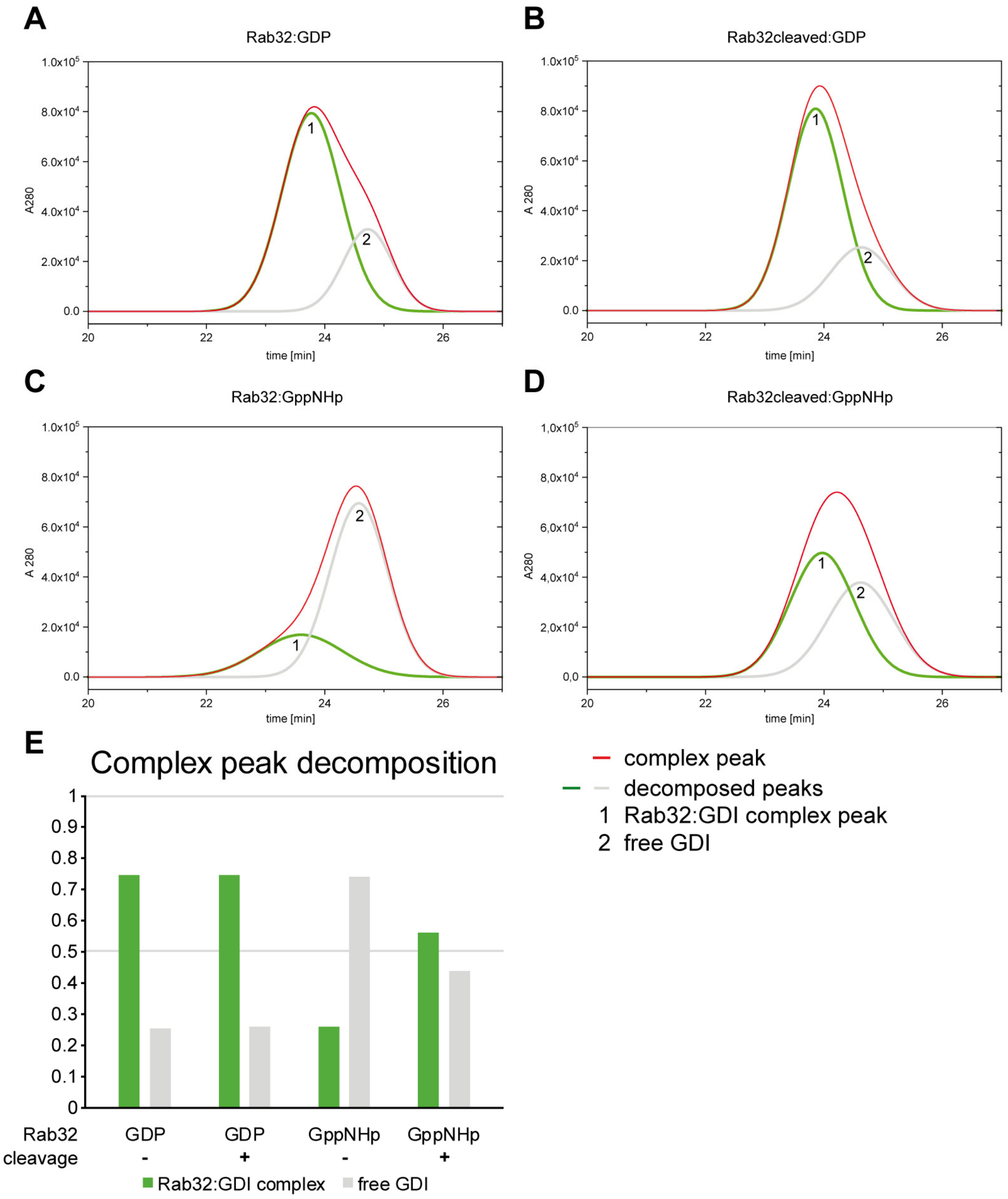

Figure S3: Decomposition of eluted complex peaks of Rab32 and GDI using aSEC. Related to Figure 2C and 2D. (**A**) Rab32:GPD:GDI, (**B**) $Rab32_{cleaved}$:GDP:GDI, (**C**) Rab32:GppNHp:GDI, and (**D**) $Rab32_{cleaved}$:GppNHp:GDI showing both the absorption at 280 nm (red chromatogram) as well as the decomposition of the peak into free GDI (2) and the Rab32:GDI complex (1). Peak decomposition was performed using Origin data analysis software (OriginLab 2019b, v9.65) setting the elution time of free GDI in complex peak to 24.6 min. (**E**) The populations determined in panels A-D are plotted as a bar chart.

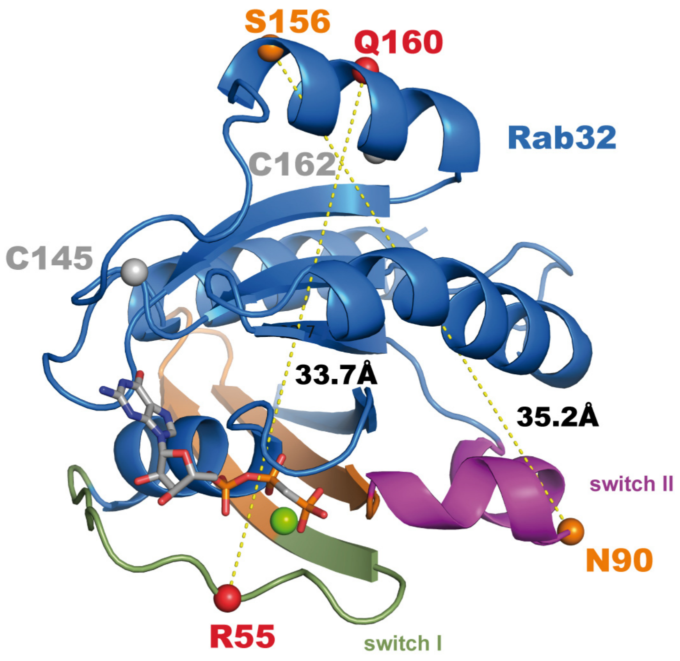

Figure S4: Positions of mutated Cys-residues within the Rab32 structure. Related to Figure 3A. The positions of cysteine mutations and the distances between them are indicated by yellow dotted lines for the two spFRET pairs used (red and orange spheres). Further wild type Cys residues (grey spheres) were mutated to Ser. The positions were labelled stochastically with donor and acceptor molecules. (PDB: 6FF8 (McGrath et al., 2019)).

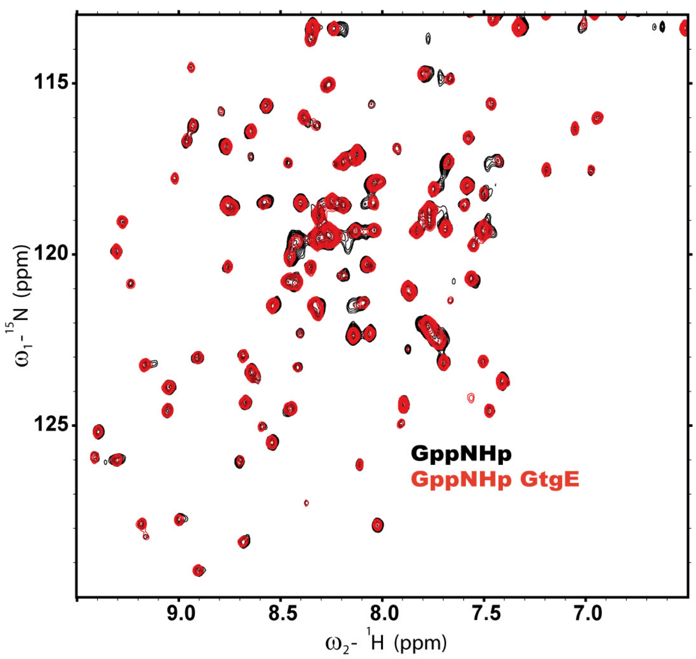

Figure S5: ^{1}H ^{15}N HSQC spectra of Rab32:GppNHp with GtgE supplementation. Related to Figure 4. Superimposed spectra of Rab32:GppNHp and Rab32:GppNHp in the presence of GtgE protease are very similar. No significant differences in the signals were observed indicating that nucleotide exchange with GppNHp *in vitro* was already fully completed.

Table S1: Anisotropy data of utilized fluorophores in Rab32 spFRET measurements. Related to Figure 3C and 3D. Residual fluorescence anisotropy r_{inf} and steady-state fluorescence anisotropy r_{ss} of Alexa488 as the donor and Alexa647 as the acceptor are given.

	**Donor only**		**Acceptor**	
**Rab32 mutant**	r_{inf}	r_{ss}	r_{inf}	r_{ss}
**R55C Q160C**	0.070	0.081	0.077	0.10
**R55C Q160C cleaved**	0.085	0.10	0.20	0.29
**N90C S156C**	0.081	0.12	0.17	0.21
**N90C S156C cleaved**	0.11	0.12	0.21	0.28

Transparent Methods

Molecular Biology

All cloning procedures were accomplished using the Mach1 *E.coli* strain. Q5® Site-Directed Mutagenesis Kit (KLD, New England Biolabs) was used for generation of all mutants used for this study according to a provided protocol by the manufacturer. The site and ligation independent cloning (SLIC) strategy was applied for generation of all plasmids containing wild type genes used in this study. Rab32 constructs, SopD2 and GtgE were cloned into the pMal™-c2X vector (NEB) with the Factor Xa site replaced by a TEV cleavage site. GDI was cloned into a pFastBacHT A vector for following usage in insect expression system (Oesterlin et al., 2012). Proteins were designed as N-terminal His_6-MBP (and just His_6 with GDI)-fusion proteins. The tags were separated from the gene of interest by a TEV protease cleavage site. Rab32 applied in GDI interaction studies contained CVIM as a C-terminal FTase recognition sequence for prenylation. The VARP gene template was kindly provided by David Owen's Lab (University of Cambridge; (Hesketh et al., 2014)). All further wild type constructs used in this work were generated in a previous study (Wachtel et al., 2018). Oligonucleotides were purchased from Integrated DNA Technologies, Inc. (IDT, Leuven, Belgium). All plasmids were confirmed by Sanger sequencing with Microsynth Seqlab. NCBI accession numbers or Uniprot IDs for the proteins used in this study: Rab32 variants: $Rab32_{1\text{-}225}$, $Rab32_{20\text{-}201}$, $Rab32_{20\text{-}227(CVIM)}$ (NP_006825), VARP-$ANK1_{451\text{-}640}$ (NP_115515), $GDI_{1\text{-}447}$ (P21856), $SopD2_{1\text{-}319}$ (Q8ZQC8), $GtgE_{1\text{-}228}$ (A0A0H3N9Y3).

Protein expression

For recombinant production of proteins, the *E. coli* BL21-Codon Plus (DE3) strain (for the Rab32 and the VARP-ANK1 fusion proteins) or BL21-Codon Plus (DE3)-RIL strain (for GtgE and SopD2) were transformed with 100 ng of the respective plasmid and grown overnight in 20 ml of lysogeny broth (LB) medium containing 125 µg/mL ampicillin (and 34 µg/mL chloramphenicol for BL21-Codon Plus (DE3)-RIL cells) at 37°C and 180 rpm (Innova 44 shaking incubator, New Brunswick). The expression cultures (1 L of LB medium or ^{15}N supplemented M9 minimal medium for isotope labelling) containing corresponding antibiotics were inoculated with 20 ml of the overnight cultures and grown under same conditions. At OD_{600} = 0.5-0.8, protein expression was induced by addition of 1 mM isopropylthiogalactopyranosid (IPTG, final) for Rab32 variants and VARP-ANK1 as well as 0.5 mM IPTG for GtgE and SopD2, followed by overnight incubation at 22°C and 180 rpm. GDI was expressed using the baculovirus expression system in *S. frugiperda* cells (Sf9, Thermo Scientific) and was purified as described before (Oesterlin et al., 2012). Cells were harvested by centrifugation at 8500 g and 20°C for 30 min (Sigma 8K, Sigma Centrifuges). After resuspending and washing the pelleted bacteria in phosphate buffered saline (PBS, 137 mM

NaCl, 2.7 mM KCl, 10 mM NaH_2PO_4, 2 mM KH_2PO_4), cells were centrifuged at 3000 g and 4°C for 20 min (5810 R, Eppendorf). The pellets were snap frozen in liquid nitrogen and stored at -20°C until further use.

Protein purification

Pellets containing GtgE, SopD2 or VARP-ANK1 were resuspended in buffer A (50 mM HEPES, 500 mM NaCl, 1 mM $MgCl_2$, 2 mM β-Mercaptoethanol (ß-ME) at pH 7.5 in a ratio of 10 ml buffer to 1 g pellet. For pellets containing Rab32 buffer A was supplemented with 10 µM Guanosine 5′-diphosphate (GDP). A spatula tip of DNAse I (Sigma Aldrich) was added to the suspension and cells were disrupted in a high-pressure fluidizer at 1.8 kbar (Constant Systems Ltd.). Subsequently, 1 mM phenylmethylsulfonylfluorid (PMSF, final concentration) was added and the crude protein extract was cleared by centrifugation at 20000 rpm and 4°C for 45 min (Avanti J-26 XP with JA25.50 rotor, Beckman Coulter). The crude lysate containing VARP-ANK1 was supplemented with 1 mM 4-(2-Aminoethyl)benzenesulfonyl fluoride (AEBSF; Sigma Aldrich, final concentration) and 5% of glycerol (v/v) before clearing the lysate.

For the purification of all proteins via fused His_6-affinity tag, the cleared protein extract was applied to an immobilized metal affinity chromatography (IMAC) using a 5 mL Nuvia column chelating Ni^{2+}-ions (Bio-Rad Laboratories) that was equilibrated with buffer A beforehand. For all chromatography purifications, the NGC Quest^{TM} 10 medium-pressure liquid chromatography (MPLC) system (Bio-Rad) was used. The cleared lysate was loaded onto the column and washed with 6-8 column volumes (CV) 8% buffer B (buffer A supplemented with 500 mM imidazole). Subsequently, a 20 CV gradient of 8-100% buffer B was applied, where target proteins eluted between 18-35% buffer B. Relevant fractions were analyzed by sodium dodecylsulfate polyacrylamide gel electrophoresis (SDS-PAGE) and pure protein fractions were pooled. Subsequently, the proteins were dialyzed against 5 L dialysis buffer (20 mM HEPES, 100 mM NaCl, 2 mM ß-ME at pH 7.5) overnight at 4°C. During dialysis, the target proteins were incubated with 1 mg TEV-protease (containing a His_7-affininty tag, in house preparation) per 40 mg target protein in order to quantitatively cleave the fused His_6-affinity and MBP solubility tag. To purify the proteins of interest from the protease and the cleaved tags, reverse IMAC was applied, either collecting the target protein in the flow through (GtgE, SopD2, and VARP-ANK1) or 5% buffer B elution fraction (Rab32 variants).

For further purification size exclusion chromatography using a 16/600 Superdex 75 pg column (GE Healthcare) was performed. The column was equilibrated with gel filtration buffer (20 mM HEPES, 50 mM NaCl, 1 mM $MgCl_2$, 10 µM GDP, 2 mM DTT at pH 7.5 for Rab32 and the same buffer system without GDP for other proteins). Fractions containing pure and monodispers protein of interest were identified by SDS-PAGE, concentrated to 5-30 mg/mL using Amicon Ultra 15 ml centrifugal filters (Merck Millipore), flash frozen in liquid nitrogen, and stored at -80°C.

GTPase nucleotide loading

The inactive Rab32:GDP variants were prepared by incubation of the crude protein extract with purified, tag-free SopD2 (50 nM) directly after cell disruption for 30 min at room temperature before the first IMAC was performed. The GTP or GppNHp exchange was performed in SEC buffer by supplementing the buffer with 5 mM EDTA and 40-fold excess of the desired nucleotide to a small GTPase and incubated at room temperature for 2 h. Nucleotide exchange was stopped by adding 5 mM $MgCl_2$ (final concentration) to the protein solution and separation of the GTPase from excess of nucleotide was performed using a NAP5 desalting column equilibrated with SEC buffer containing 10 µM GDP or 1 µM GppNHp respectively. Protein containing eluate was confirmed with NanoDrop™ 2000 (Thermo Scientific), flash frozen in liquid nitrogen, and stored at -80°C. Nucleotide loading efficiency was verified by ion-pairing reversed-phase high performance chromatography. To this end, protein samples (40 µM, 22 µL) were heat precipitated at 95°C for 5 min and centrifuged for 5 min at 21000 g. Supernatant was subjected to chromatographic separation on Shimadzu UFPLC (Prominence series) equipped with C18 column (Prontosil C18, F184PS050, Bischoff Chromatography) using 50 mM potassium phosphate buffer pH 6.6, 10 mM tetra-n-butylammonium bromide, 12% acetonitrile (v/v). Nucleotides were detected at 254 nm and resulting peaks were integrated and normalized to the total amount of nucleotides detected set to 100%. The retention time of each nucleotide was determined with the respective nucleotide standard in a separate run.

Rab32 farnesylation

Rab32 was incubated with FTase (in house preparation as described before (Hougland et al., 2012)) in the presence of farnesyl pyrophosphate (FPP, Sigma Aldrich) for 2 h at 30°C and 300 rpm (Rab32:FTase:FPP molar ratio 2:1:12) in the reaction buffer (20 mM HEPES, 50 mM NaCl, 1 mM $MgCl_2$, 0.5 mM $ZnCl_2$, 10 µM GDP, 2 mM DTT at pH 7.5). Farnesylation was confirmed via intact protein mass spectrometry on a LCMS system.

Rab32 proteolysis by GtgE

For quantitative modification, Rab32:GDP was submitted to GtgE-mediated proteolysis at 25°C for 2 h (GtgE:Rab32 molar ratio 1:200) in a gel filtration buffer (20 mM HEPES, 50 mM NaCl, 1 mM $MgCl_2$, 10 µM GDP, 2 mM DTT at pH 7.5). Cleaved Rab32 variants used in spFRET measurements were generated as described recently (Fauser et al., 2020). Proteolysis completion was monitored via SDS-PAGE.

Protein complex formation on analytical size exclusion chromatography

For the complex formation of Rab32 with VARP-ANK1 and GDI a Superdex 75 10/300 GL (GE Healthcare) was attached to a UFPLC system (Prominence series, Shimadzu). For complex formation with VARP, the respective Rab32 preparation was incubated with VARP-ANK1 in a

1:1 molar ratio (50 µM) at 15°C for 1 hour. For GDI binding studies, Rab32 was additionally farnesylated before the incubation with GDI for complex formation (1:1 molar ratio, 50 µM). Subsequently, 50 µl were chromatographically separated on an aSEC and fractionated to 0.5 ml fractions. The gel filtration buffer (20 mM HEPES at pH 7.5, 50 mM NaCl, 1 mM $MgCl_2$, 2 mM DTT, 10 µM GDP or 1 µM GppNHp respectively) was used as a mobile phase for protein separation at 0.5 ml/min flow rate. Proteins were detected at 280 nm. The individual runs of single proteins served as a reference. Vitamin B_{12} was used as an internal standard.

Fluorescent labeling of proteins

The buffer of the proteins was exchanged to 20 mM HEPES, 50 mM NaCl, 1 mM $MgCl_2$, 10 µM GDP at pH 7.5 using a PD MiniTrap G-25 column (GE Healthcare). After concentrating the protein with a Vivaspin 500 10 kDa (Sartorius) spin column, the protein was added to a premixed dye solution (2x molar excess of Alexa488-Maleimide and Alexa647-Maleimide) and incubated at room temperature for 2 h. The remaining free dye was removed using a PD MiniTrap G-25 column and the protein was concentrated using Vivaspin 500 10 kDa (Sartorius) centrifugal filters.

Single-pair FRET measurements

For the spFRET measurements, the Rab32 mutants were diluted to concentrations in the range of 20–100 pM in buffer containing 20 mM HEPES, 50 mM NaCl, 1 mM $MgCl_2$ and 10 µM GDP at pH 7.5. Measurements were carried out on a homebuilt (Barth et al., 2019) three-color dual-polarization confocal microscope with pulsed-interleaved excitation (Kapanidis et al., 2004; Müller et al., 2005) and multi-parameter fluorescence detection (Eggeling et al., 2001) using only two of the laser lines (LDH-D-C-485, LDH-D-C-640, PicoQuant). The laser power measured before the objective were 100 µW for blue excitation and 70 µW for red excitation. Bursts of single molecules diffusing through the confocal volume of the microscope were measured for at least 3 h. The obtained data was analyzed with PAM (Schrimpf et al., 2018), a Matlab-based software. Bursts with a minimum of 100 photons were selected and the ALEX-2CDE filter (Tomov et al., 2012) was used to further narrow down the burst selection to only double-labeled proteins. Single-dye populations were used to calculate crosstalk and direct excitation. The γ-factor was calculated by a linear interpolation of 1/S vs. E of a control measurement of a DNA sample containing two different FRET populations, since the protein measurements mainly showed only one population. Distances were determined using the photon distribution analysis (Antonik et al., 2006) with a Förster radius of 52 Å, which was adjusted from the manufacturers value due to our assumption of $n = 1.4$ for the index of refraction and the decreased lifetime and thus reduced quantum yield of Alexa488 when labeled to Rab32.

Molecular dynamics simulations

The crystal structure of Rab32 in complex with GppCp and the effector VARP (PDB:4CYM (Hesketh et al., 2014)) served as the starting structure for the molecular dynamics simulations. VARP atoms were removed and the carbon atom between the β- and ɣ-phosphate was replaced with oxygen to replicate GTP. Furthermore, a Mg^{2+} ion was inserted in a position such that it had contact with both the β- and ɣ-phosphate. The gamma phosphate was removed to form the GDP-bound variants of Rab32. The Amber ff14sb force field (Maier et al., 2015) was used for the protein, while additional parameters for GDP and GTP were taken from the Amber parameter database (Meagher et al., 2003). The peptide bond between G59 and V60 was removed to form the proteolized variants of Rab32. Sodium and chloride ions were added to reach a salt concentration of 0.1 M. The complex was set at the center of a truncated octahedron box large enough to have a minimum distance of 10 Å from the edges and filled with water molecules that were modelled using OPC 4-point rigid model (Izadi et al., 2014). The solvated box was then energy minimized (5000 steps), followed by 25 ps of heating and 50 ps of density equilibration, followed by a simulation using an NPT ensemble at 300 K. During these phases, the protein's heavy atoms, the nucleotide and the magnesium ions were restrained at their initial positions using a harmonic potential with a decreasing force constant starting at 5.0 $kcal.mol^{-1}Å^{-2}$ and ending with 1.0 $kcal.mol^{-1}Å^{-2}$. The rest of the simulations were performed without any restraints. The pmemd version of the Amber 16 software package was used employing the hydrogen mass repartitioning feature of the Parmed tool, which allows a simulation time step of 4fs (Case et al., 2016). Long range interactions were included using the particle mesh Ewald (PME) method combined with periodic boundary conditions and a 9 Å cut-off for real space non-bonded interactions. Trajectories were processed and analyzed using CPPTRAJ program. Figures were generated using the PyMol software package (Schrödinger, 2015).

NMR

The uniformly ^{15}N-labeled NMR samples of Rab32 were at 100 µM protein concentration in buffer (20 mM HEPES pH 7.5, 50 mM NaCl, 1 mM $MgCl_2$, 2 mM DTT, 10 µM GDP) with 10% D_2O for a lock signal. NMR experiments were recorded at 298 K on a 600-MHz Bruker Avance NMR spectrometer with cryogenic triple resonance gradient probes. NMR spectra were processed by TOPSPIN3.5 (Bruker), then analyzed using NMRFAM-SPARKY (Lee et al., 2015). Due to the limited sample stability and quantity, our multiple attempts toward signal assignment were not successful.

Circular dichroism

Measurements of CD spectra were carried out on a JASCO 715 CD spectrometer equipped with a Peltier-temperature controller. Spectra were measured at 20 µM protein concentration

in a quartz cuvette with 1 mm path length (Hellma) in 1 mM HEPES pH 7.5, 2.5 mM NaCl, 50 µM $MgCl_2$, 0.5 µM GDP or GppNHp. The temperature was increased by 30°C/h and molar ellipticity was measured at 220 nm. Data were normalized by setting the plateau signal of the thermally unfolded protein to 1.0.

Fluorescence spectroscopy

For determination of the nucleotide dissociation kinetics of Rab32 and $Rab32_{cleaved}$, appropriate GTPase was loaded with GDP or GppNHp (not hydrolysable GTP analogue) bearing a mant fluorescent moiety. The mant-GDP or mant-GppNHp nucleotide dissociation was monitored via the release of the fluorescent nucleotide resulting in a decrease of the fluorescent signal using a fluorescence spectrometer FP-8300 (JASCO) with the following parameters: λ_{exc}: 360 nm, λ_{em}: 440 nm, excitation slit: 1 nm, emission slit: 5 nm. Measurements were conducted at 20°C in 20 mM HEPES, 50 mM NaCl, 1 mM $MgCl_2$, 1 mM ß-ME at pH 7.5 with 500 nM Rab32. Nucleotide exchange was started by addition of an excess of the corresponding mant-free nucleotide (400x) to the Rab32 in the cuvette (Hellma). Full dissociation was achieved by addition of 5 mM EDTA into the cuvette to maximize the nucleotide release.

Determination of mant-nucleotide dissociation rates

For the determination of nucleotide dissociation (k_{off}) measured by fluorescence spectroscopy, reaction curves were fitted to a one-phase exponential decay function with time constant parameter according to equation 1 using the software OriginPro (OriginLab, 2019b, v9.65).

$$F(t) = F_0 + F_A \cdot e^{-t \times k_{off}} \quad \text{equation (1)}$$

with F(t): fluorescence intensity, F_0: minimum fluorescence intensity, F_A: total fluorescence amplitude (i.e. $F_{max} - F_0$, with F_{max}: maximum fluorescence intensity), k_{off}: dissociation constant. Statistical significance was validated by paired Student's t-test in GraphPad Prism (Version 4.0).

Mass spectrometry

All samples for mass spectrometry (MS) analysis were diluted in water to a final protein concentration of 0.1 mg/ml and 1 µl was applied to an ESI-ion trap mass spectrometer (LCQ fleet, Thermo Scientific) coupled to an UHPLC system (Ultimate 3000, Thermo Scientific) equipped with a ProSwift™ RP-4H column (1×50 mm, Thermo Scientific) at a flow rate of 0.2 ml/min. The proteins eluted with a linear gradient of 5–50% acetonitrile (0.1% formic acid) in 6 min. The total ion chromatogram raw data were analyzed with the Xcalibur Software v.3.1 (Thermo Scientific) and deconvoluted using MagTran v.1.02 (Zhang and Marshall, 1998).

References

Antonik, M., Felekyan, S., Gaiduk, A., Seidel, C.A.M., 2006. Separating structural heterogeneities from stochastic variations in fluorescence resonance energy transfer distributions via photon distribution analysis. J Phys Chem B *110*, 6970–6978.

Barth, A., Voith von Voithenberg, L., Lamb, D.C., 2019. Quantitative Single-Molecule Three-Color Förster Resonance Energy Transfer by Photon Distribution Analysis. J Phys Chem B *123*, 6901–6916.

Case, D.A., Betz, R.M., Cerutti, D.S., Cheatham, T.E., III, Darden, T.A., Duke, R.E., Giese, T.J., Gohlke, H., Goetz, A.W., Homeyer, N., Izadi, S., Janowski, P., Kaus, J., Kovalenko, A., Lee, T.S., LeGrand, S., Li, P., Lin, C., Luchko, T., Luo, R., Madej, B., Mermelstein, D., Merz, K.M., Monard, G., Nguyen, H., Nguyen, H.T., Omelyan, I., Onufriev, A., Roe, D.R., Roitberg, A., Sagui, C., Simmerling, C.L., Botello-Smith, W.M., Swails, J., Walker, R.C., Wang, J., Wolf, R.M., Wu, X., Xiao, L. and Kollman, P.A., 2016. AMBER 2016, University of California, San Francisco.

Eggeling, C., Berger, S., Brand, L., Fries, J.R., Schaffer, J., Volkmer, A., Seidel, C.A.M., 2001. Data registration and selective single-molecule analysis using multi-parameter fluorescence detection. Journal of Biotechnology *86*, 163–180.

Fauser, J., Savitskiy, S., Fottner, M., Trauschke, V., Gulen, B., 2020. Sortase-Mediated Quantifiable Enzyme Immobilization on Magnetic Nanoparticles. Bioconjug Chem *31*, 1883–1892.

Hesketh, G.G., Pérez-Dorado, I., Jackson, L.P., Wartosch, L., Schäfer, I.B., Gray, S.R., McCoy, A.J., Zeldin, O.B., Garman, E.F., Harbour, M.E., et al., 2014. VARP is recruited on to endosomes by direct interaction with retromer, where together they function in export to the cell surface. Dev Cell *29*, 591–606.

Hougland, J.L., Gangopadhyay, S.A., Fierke, C.A., 2012. Expansion of protein farnesyltransferase specificity using "tunable" active site interactions: development of bioengineered prenylation pathways. J Biol Chem *287*, 38090–38100.

Izadi, S., Anandakrishnan, R., Onufriev, A.V., 2014. Building Water Models: A Different Approach. J Phys Chem Lett *5*, 3863–3871.

Kapanidis, A.N., Lee, N.K., Laurence, T.A., Doose, S., Margeat, E., Weiss, S., 2004. Fluorescence-aided molecule sorting: analysis of structure and interactions by alternating-laser excitation of single molecules. Proc Natl Acad Sci U S A *101*, 8936–8941.

Lee, W., Tonelli, M., Markley, J.L., 2015. NMRFAM-SPARKY: enhanced software for biomolecular NMR spectroscopy. Bioinformatics *31*, 1325–1327.

Maier, J.A., Martinez, C., Kasavajhala, K., Wickstrom, L., Hauser, K.E., Simmerling, C., 2015. ff14SB: Improving the Accuracy of Protein Side Chain and Backbone Parameters from ff99SB. J Chem Theory Comput *11*, 3696–3713.

McGrath, E., Waschbüsch, D., Baker, B.M., Khan, A.R., 2019. LRRK2 binds to the Rab32 subfamily in a GTP-dependent manner via its armadillo domain. Small GTPases, 1–14.

Meagher, K.L., Redman, L.T., Carlson, H.A., 2003. Development of polyphosphate parameters for use with the AMBER force field. J Comput Chem *24*, 1016–1025.

Müller, B.K., Zaychikov, E., Bräuchle, C., Lamb, D.C., 2005. Pulsed interleaved excitation. Biophys J *89*, 3508–3522.

Oesterlin, L.K., Goody, R.S., Itzen, A., 2012. Posttranslational modifications of Rab proteins cause effective displacement of GDP dissociation inhibitor. Proc Natl Acad Sci U S A *109*, 5621–5626.

Schrimpf, W., Barth, A., Hendrix, J., Lamb, D.C., 2018. PAM: A Framework for Integrated Analysis of Imaging, Single-Molecule, and Ensemble Fluorescence Data. Biophys J *114*, 1518–1528.

Schrödinger, 2015. LLC, The PyMOL Molecular Graphics System, Version 2.0.

Tomov, T.E., Tsukanov, R., Masoud, R., Liber, M., Plavner, N., Nir, E., 2012. Disentangling subpopulations in single-molecule FRET and ALEX experiments with photon distribution analysis. Biophys J *102*, 1163–1173.

Wachtel, R., Bräuning, B., Mader, S.L., Ecker, F., Kaila, V.R.I., Groll, M., Itzen, A., 2018. The protease GtgE from Salmonella exclusively targets inactive Rab GTPases. Nat Commun *9*, 44.

Zhang, Z., Marshall, A.G., 1998. A universal algorithm for fast and automated charge state deconvolution of electrospray mass-to-charge ratio spectra. J Am Soc Mass Spectrom *9*, 225–233.
